# Proteomics analysis identified peroxiredoxin 2 involved in early-phase left ventricular impairment in hamsters with cardiomyopathy

**DOI:** 10.1371/journal.pone.0192624

**Published:** 2018-02-13

**Authors:** Kentaro Kuzuya, Sahoko Ichihara, Yuka Suzuki, Chisa Inoue, Gaku Ichihara, Syota Kurimoto, Shinji Oikawa

**Affiliations:** 1 Department of Human Functional Genomics, Life Science Research Center, Mie University, Tsu, Japan; 2 Graduate School of Regional Innovation Studies, Mie University, Tsu, Japan; 3 Department of Occupational and Environmental Health, Tokyo University of Science, Noda, Japan; 4 Department of Environmental and Molecular Medicine, Mie University Graduate School of Medicine, Tsu, Japan; Emory University, UNITED STATES

## Abstract

Given the hypothesis that inflammation plays a critical role in the progression of cardiovascular diseases, the aim of the present study was to identify new diagnostic and prognostic biomarkers of myocardial proteins involved in early-phase cardiac impairment, using proteomics analysis. Using the two-dimensional fluorescence difference gel electrophoresis (2D-DIGE) combined with MALDI-TOF/TOF tandem mass spectrometry, we compared differences in the expression of proteins in the whole left ventricles between control hamsters, dilated cardiomyopathic hamsters (TO-2), and hypertrophy cardiomyopathic hamsters (Bio14.6) at 6 weeks of age (n = 6, each group). Proteomic analysis identified 10 protein spots with significant alterations, with 7 up-regulated and 3 down-regulated proteins in the left ventricles of both TO-2 and Bio 14.6 hamsters, compared with control hamsters. Of the total alterations, peroxiredoxin 2 (PRDX2) showed significant upregulation in the left ventricles of TO-2 and Bio 14.6 hamsters. Our data suggest that PRDX2, a redox regulating molecule, is involved in early-phase left ventricular impairment in hamsters with cardiomyopathy.

## Introduction

Cardiomyopathy leads to serious congestive heart failure (CHF), which is the main cause of mortality and morbidity in children and adults [1]. Cardiomyopathy is classified mainly into hypertrophic and dilated cardiomyopathies (HCM and DCM, respectively) based on the gross appearance of the heart. The pathology of these cardiomyopathies is overlapping and common genetic mutations exist [2]. However, the pathogenetic relationship between the causative genes is not well understood. A better understanding of the pathogenetic interplay between each causative gene is crucial to design novel therapeutic strategies for cardiomyopathy. For this purpose, an animal model of hereditary cardiomyopathy is extremely useful [3,4]. Distinct sublines of Syrian hamster manifesting HCM (Bio-14.6) or DCM (TO-2) have been established from the original line Bio-1.50 [5]. Bio14.6 hamster exhibits marked cardiac hypertrophy in its early stage. On the other hand, the left ventricular (LV) wall of the TO-2 hamster is very thin and has a shorter life expectancy than the Bio-14.6 hamster. It has been reported that the genetic cause of Bio14.6 hamster is a mutation of the gene encoding delta-sarcoglycan (δ-SG), one of the dystrophin-associated proteins [6, 7]. It has been demonstrated also that TO-2 hamster shares with Bio-14.6 hamster the same deletion of the genomic interval that spans about 30 kilobases and includes the two promoters of the δ-SG gene [7, 8]. These findings indicate the presence of a common genetic modifier in cardiomyopathic hamsters that might be involved in compensatory cardiac hypertrophy or cardiac dysfunction [9].

The field of proteomics provides a systematic approach for quantitative and qualitative mapping of the whole proteome [10]. Proteomics analysis is used to complete complement of proteins expressed by a biological system under different physiological or pathological conditions [11]. Since some people who have cardiomyopathy present with serious symptoms and complications [12], new methods for early diagnosis and new therapeutic alternatives for cardiomyopathy are necessary. Based on the concept that examination of changes in the proteome offers insight into cellular and molecular mechanisms [13], the present study was designed to apply proteomics analysis to determine changes in the protein profile in cardiomyopathy. Specifically, we determined the changes in the expression of proteins in the cardiac tissues of cardiomyopathy in order to find possible molecular mechanisms of cardiac impairment in cardiomyopathy.

## Methods

### Experimental animals

Male TO-2 and Bio14.6 cardiomyopathic Syrian hamsters and control F1B hamsters were obtained from BIO Breeders (Fitchburg, MA) [14]. Hamsters were sacrificed at 6 weeks of age. The investigation conformed to the Guide for the Care and Use of Laboratory Animals published by the US National Institutes of Health (NIH Publication No. 85–23, revised 1996) and was approved by the Committee on Laboratory Animals Utilization of Mie University.

### Physiological measurements

Transthoracic echocardiography was performed before sacrifice, using the HD 11 XE system (Philips Medical Systems, Bothell, WA) by intraperitoneal injection of 50 mg/kg body weight of pentobarbital sodium [15]. Wall thickness and left ventricular (LV) end-diastolic diameter were obtained from the short-axis view at the level of the papillary muscles, and LV fractional shortening was calculated.

### Reverse transcription-polymerase chain reaction (RT-PCR) analysis

Total RNA was extracted from heart tissue (n = 6 per group) using the ReliaPrep RNA Tissue Miniprep System (Promega, Madison, WI), according to the instructions provided by the manufacturer. Then, total RNA was subjected to quantitative RT-PCR analysis with primers specific for mRNAs encoding atrial natriuretic peptide (ANP), brain natriuretic peptide (BNP), collagen I, and collagen III genes, using ABI PRISM 7000 Sequence Detection System (Applied Biosystems, Foster City, CA). All primers were shown in S1 Table. Standard curves were generated for each primer set and a coefficient file generated to quantify the expression of each gene relative to glyceraldehyde-3-phosphate dehydrogenase (GAPDH). All experiments were performed in duplicates.

### Preparation of heart protein samples

The heart was carefully dissected out after sacrifice and immediately frozen in liquid nitrogen and stored at -80 °C. Frozen tissues were homogenized in lysis buffer [30 mM Tris-HCl, 7 M urea, 2 M thiourea, 4% w/v CHAPS, and cocktails of protease inhibitors (Complete Mini, EDTA-free and Pefabloc SC PLUS; Roche, Basel, Switzerland), pH 8.5]. After incubation for 60 min on ice, homogenates were centrifuged at 30,000 × *g* for 30 min at 4 °C and the supernatant was collected. The protein concentration in the supernatant was determined by the Bradford assay (Bio-Rad Laboratories, Hercules, CA), using bovine serum albumin as a standard.

### Two-dimensional fluorescence differential gel electrophoresis (2D-DIGE)

Each sample was labeled with amine-reactive cyanine dyes, Cy3 or Cy5 developed for fluorescence 2D-DIGE technology (GE Healthcare, Little Chalfont, United Kingdom). The heart tissue mixture was labeled with Cy2 to be used as an internal standard. After labeling with Cy3 or Cy5, we performed electrophoresis using each labeled sample and checked the volume of each sample with Image Quant TL (GE Healthcare) to confirm the protein amount of each sample. Then, 2D-DIGE was performed using the whole left ventricles of 6 hamsters of each group, as described previously [16]. After 2-DE, cyanine-labeled proteins were visualized directly by scanning, using a Typhoon 9400 imager (GE Healthcare) in fluorescence mode.

### Protein identification

Protein spots with *p<*0.05 and a fold change of ≥1.2 in all three comparisons were selected for further identification. The selected spots were excised manually from Coomassie Brilliant Blue (CBB) R-350 (PhastGel Blue R, GE Healthcare)-stained preparative 2-DE gels with gel spot cutter. Then, in-gel digestion of protein samples was performed using the protocol described previously [17]. The peptide mixtures were analyzed by a matrix-assisted laser desorption ionization time-of-flight tandem mass spectrometry (MALDI-TOF/TOF MS; 4800 *Plus* MALDI TOF/TOF^™^ Analyzer; Life Technologies, Carlsbad, CA) operating in positive-ion reflector mode. The excised proteins were identified through protein database search by the Paragon Method using Protein Pilot software (Life Technologies).

### Western blot analysis

Western blot analysis was conducted to confirm the results of proteomic analysis. The heart tissues collected at 6 weeks of age were homogenized in lysis buffer (T-PER reagent; Thermo Fisher Scientific, Waltham, MA). Samples (n = 6 per group) containing 12 μg cardiac proteins were separated by 12% SDS-PAGE and electroblotted onto polyvinylidenedifluoride (PVDF) membranes. The membranes were incubated with a rabbit polyclonal antibody to peroxiredoxin 2 (PRDX2) (ab59539; Abcam, Cambridge, United Kingdom) at 1:2,000 dilution. A rabbit monoclonal antibody to GAPDH at 1:2,000 dilution (14C10; Cell Signal Technology, Danvers, MA) was used as a loading control. The protein bands were visualized by ECL plus Western blotting detection system (GE Healthcare) and Quantity One v3.0 software (Bio-Rad Laboratories) was used to quantitate the band intensities. To prevent the detection as a saturated signal, we confirmed the antibody dilution concentration and exposure time of the CCD camera in the preliminary experiments. The expression level of PRDX2 was normalized relative to the level of GAPDH protein in the same tissue sample.

### Immunohistochemical analysis

Midventricular slices of 6-week-old hamsters were fixed in 4% paraformaldehyde, embedded in paraffin, and sectioned at thickness of 4 μm. The sections were subjected to immunostaining with rabbit polyclonal antibody specific for PRDX 2 (Abcam), as previously described [18].

### PANTHER analysis and mapping of protein expression

Protein ontology classification was performed by importing proteins into the protein analysis through the evolutionary relationships (PANTHER) classification system (http://www.pantherdb.org/, SRI International, Menlo Park, CA). Proteins were grouped according to their associated biological processes and molecular functions.

### Statistical analysis

Data are presented as mean±standard error of the mean (SEM). Comparisons among three groups were tested using one-way ANOVA followed by Dunnett’s multiple comparison tests using the JMP 8.0 software (SAS Institute Inc, Cary, NC). A *P* value<0.05 was considered statistically significant.

## Results

### Body weight and LV function

**Fig 1:**
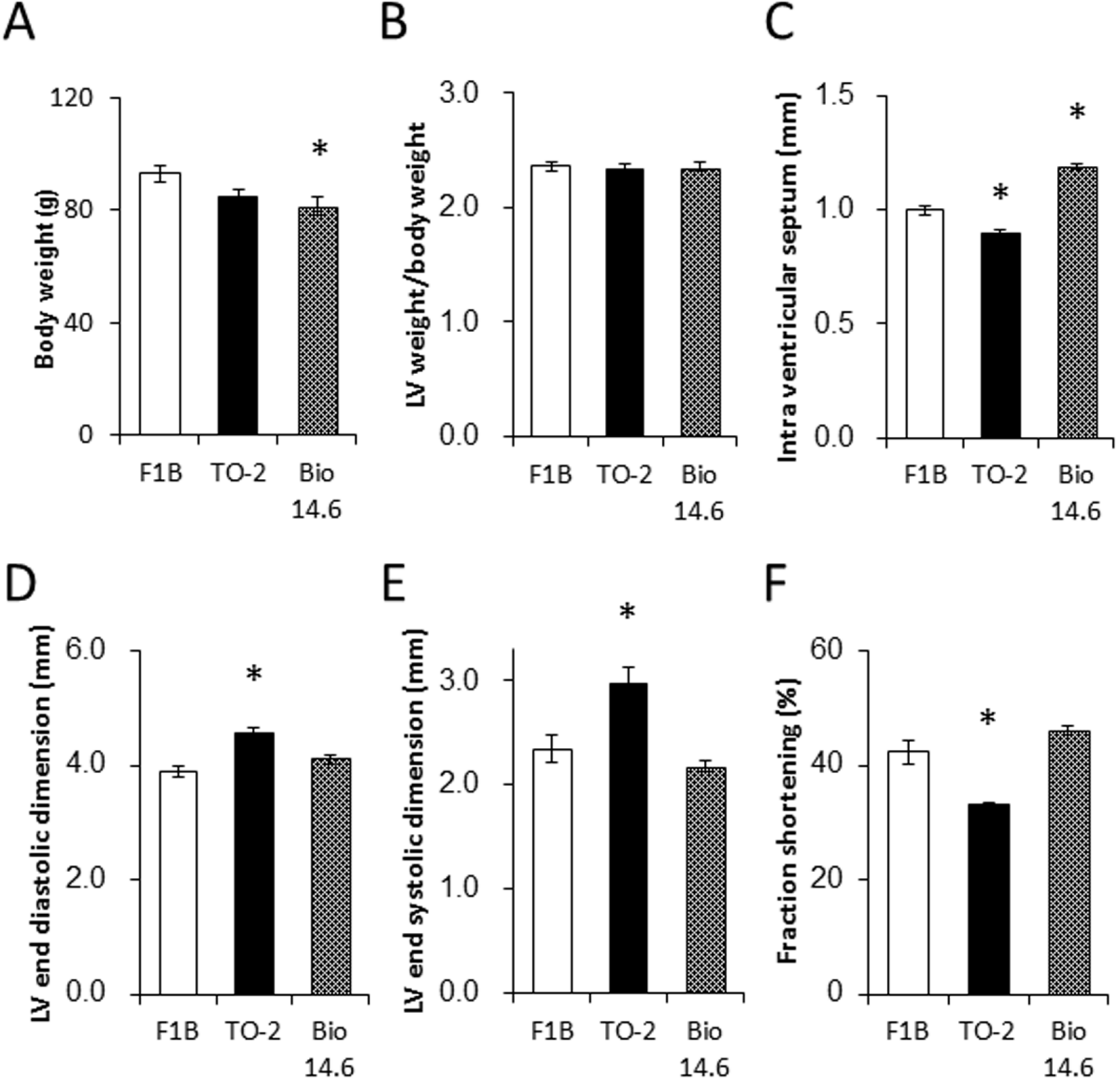
Physiological data of 6-week-old F1B, TO-2, and Bio 14.6 hamsters. (A) Body weight, (B) LV weight/body weight, (C) thickness of the intra-ventricular septum, (D) LV end-diastolic dimension, (E) LV end-systolic dimension, and (F) fraction shortening. Data are mean±SEM of six animals per group. *P<0.05 versus F1B control hamsters.

The body weight of 6-week-old Bio 14.6 hamsters was significantly lower than F1B control hamsters (Fig 1A), but there was no significant difference in body weight between F1B and TO-2 hamsters. The LV weight of Bio 14.6 hamsters was significantly lower than F1B control hamsters, but there was no significant difference in LV weight/body weight among the three strains (Fig 1B). Next, we compared the echocardiographic findings to characterize LV function. Intra ventricular septum was significantly thinner in TO-2 and significantly thicker in Bio 14.6, compared with F1B control hamsters (Fig 1C). LV end-diastolic and -systolic diameters of TO-2 hamsters were significantly larger than F1B control hamsters (Fig 1D and 1E). LV fractional shortening of TO-2 hamsters was also significantly lower than F1B control hamsters (Fig 1F). There were no significant differences in LV end-diastolic diameter, LV end-systolic diameter, and LV fractional shortening between F1B and Bio 14.6 hamsters.

### Cardiac phenotype-related gene expression

**Fig 2:**
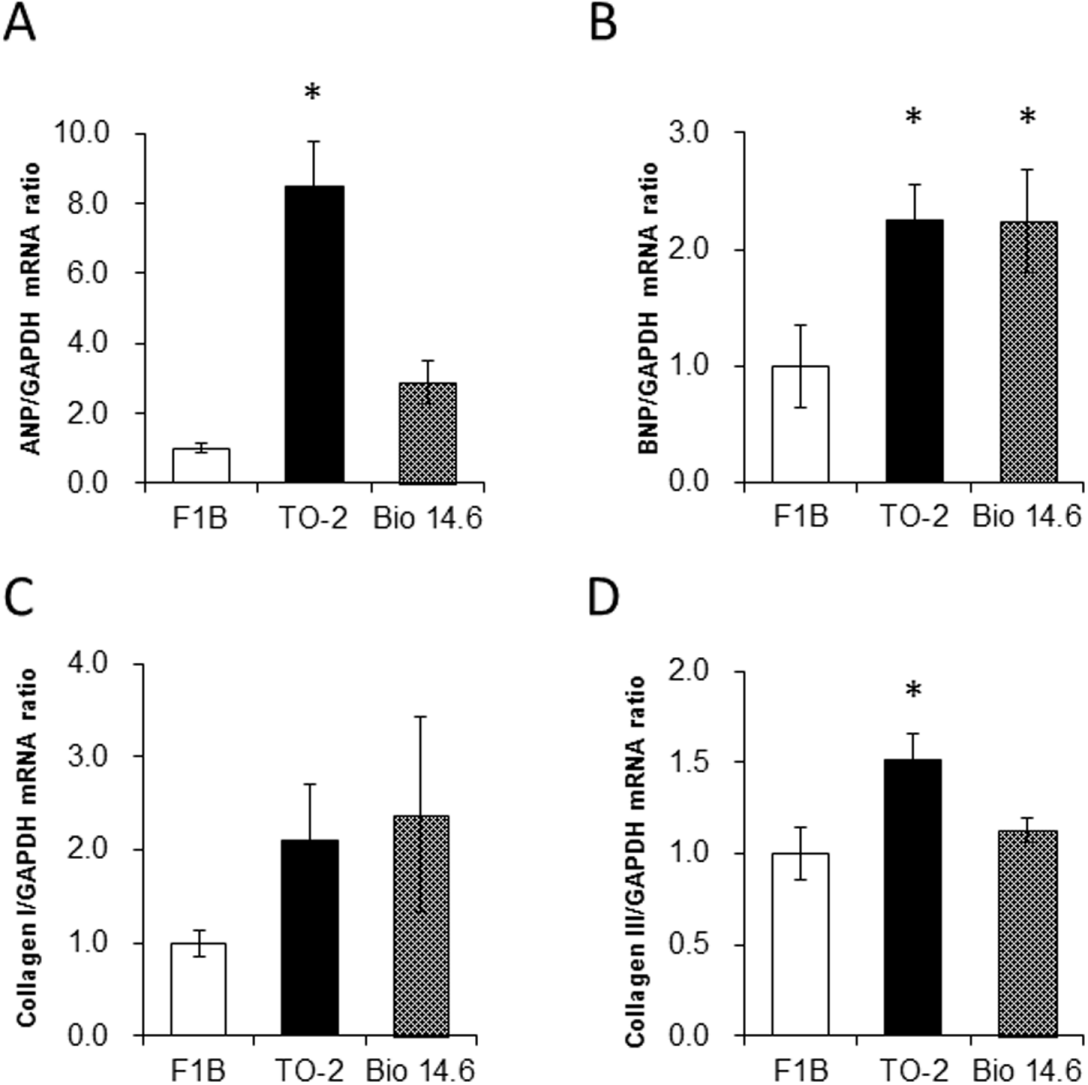
Gene expression of ANP, BNP, collagen I, and collagen III in 6-week-old F1B, TO-2, and Bio 14.6 hamsters. The mRNA levels of (A) ANP, (B) BNP, (C) collagen I, and (D) collagen III in the heart tissues were determined by quantitative RT-PCR analysis. Data are normalized by the abundance of GAPD mRNA. Quantitative data are expressed relative to the values for F1B hamsters. Data are mean s±SEM of six animals per group. *P<0.05 versus F1B control hamsters.

The abundance of ANP and collagen III mRNAs in the heart tissues was significantly greater in TO-2 hamsters than F1B control hamsters at 6 weeks of age (Fig 2A and 2D). The abundance of BNP mRNA in the heart tissues was significantly greater in both TO-2 and Bio-14.6 hamsters than in F1B control hamsters (Fig 2B). There was no significant difference in the amounts of collagen I mRNA among three hamsters (Fig 2C).

### Overall patterns of changes in protein expression

**Fig 3:**
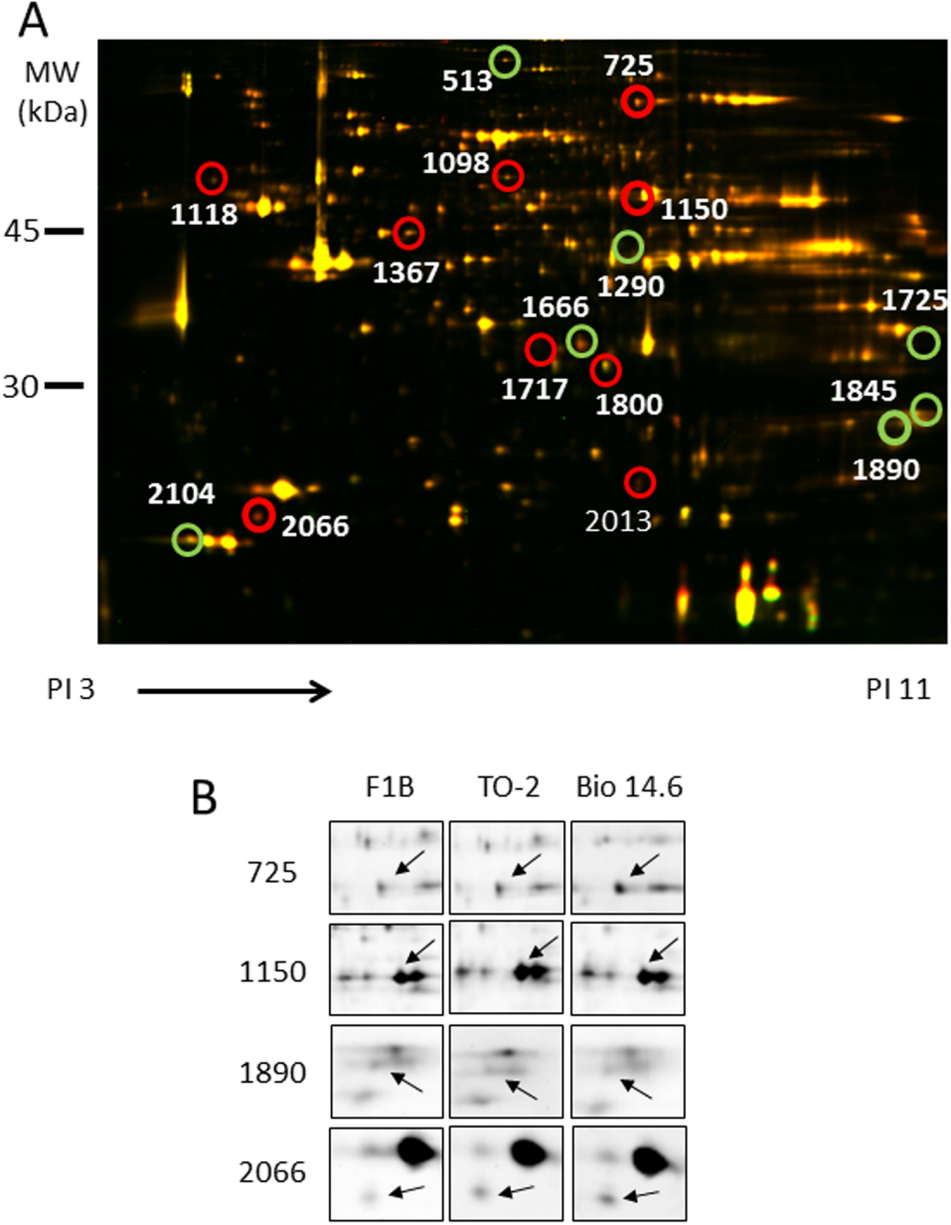
Representative 2D-DIGE image and images of spots with significant alterations in LV lysates. (A) Proteins (40 μg each) were labeled with Cy3 and Cy5 dyes, mixed and subjected to 2D-DIGE analysis. Cy3- and Cy5- images are illustrated using red and green pseudocolors, respectively. Images were analyzed using DeCyder software. IPG strips (pI 3–11) were used for IEF, and 12.5% SDS-PAGE for the second dimension. (B) The 2-DE images of 4 spots showed significant alterations in the left ventricles of both TO-2 and Bio 14.6 hamsters, compared with F1B control hamsters.

**Fig 4:**
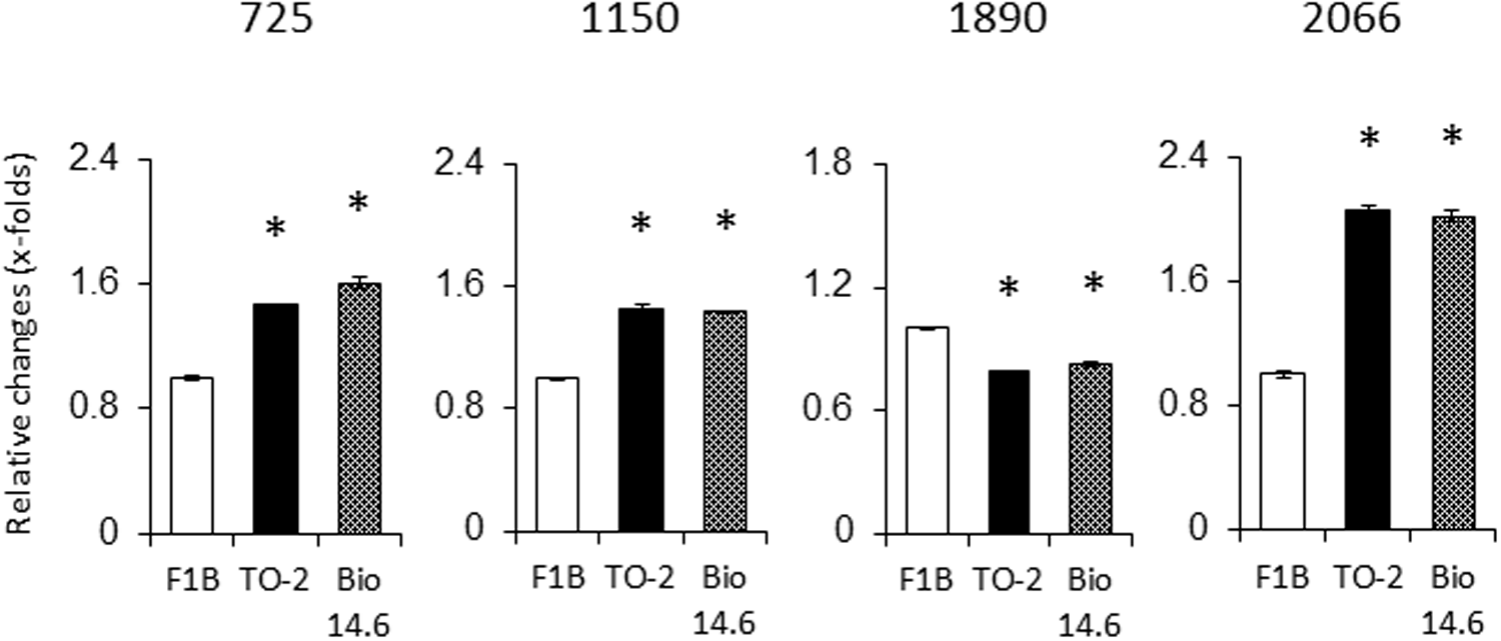
Expression levels of the identified proteins in 6 weeks-old F1B, TO-2, and Bio 14.6 hamsters. The expression levels of LV proteins with significant changes were quantified in TO-2, Bio 14.6 and F1B control hamsters. Data are mean±SEM of six animals per group. *P<0.05 versus F1B control hamsters. Numbers above each graph represented the spot number of each protein.

The proteins were extracted and subjected to comparative analysis by 2D-DIGE. Image analysis of the gels identified about 2306 spots. Fig 3A shows representative 2D-DIGE images of heart lysates of F1B and TO-2 hamsters. Proteomic analysis identified significant alterations in 10 protein spots in the left ventricles of both TO-2 and Bio 14.6 hamsters, compared with F1B control hamsters by the DeCyder software analysis, with an absolute ratio of more than 1.2-folds with statistical significance (*p*<0.05). Of the 10 spots in the 2-DE gels, 4 spots were isolated and subjected to MALDI-TOF/TOF MS (Fig 3B). Fig 4 shows the relative quantities of the 4 spots among three hamsters. These spots were identified as serotransferrin (TF) (no. 725), succinyl-CoA:3-ketoacid-coenzyme A transferase 1, mitochondrial (OXCT1) (no. 1150), phosphoglycerate mutase 2 (PGAM2) (no. 1890), and peroxiredoxin-2 (PRDX2) (no. 2066) (Table 1). With the exception of these spots, changes in 12 proteins spots in the heart were significantly different between TO-2 and F1B hamsters while changes in 6 proteins spots were significant between Bio14.6 and F1B hamsters. The 6 spots with significant differences between TO-2 and F1B hamsters were identified as creatine kinase B-type (CKB) (no. 1367), malate dehydrogenase, cytoplasmic (MDH1) (no. 1666), 3-mercaptopyruvate sulfurtransferase (MPST) (no. 1717), cytochrome c1, heme protein, mitochondrial (CYC1) (no. 1800), troponin I, cardiac muscle (TNNI3) (no. 1845), and glutathione S-transferase P (GSTP1) (no. 2013) (Table 1). Furthermore, the 6 spots with significant changes between Bio 14.6 and F1B hamsters were identified as vinculin (VCL) (no. 513), epoxide hydrolase 2 (EPHX2) (no. 1098), protein disulfide-isomerase (P4HB) (no. 1118), pyruvate dehydrogenase E1 component subunit alpha, somatic form, mitochondrial (PDHA1) (no. 1290), LIM domain-binding protein 3 (LDB3) (no. 1725), and myosin regulatory light chain 2, ventricular/cardiac muscle isoform (MYL2) (no. 2013) (Table 1).

**Table 1:** List of proteins with significant differences in their levels in heart tissues of TO-2, Bio 14.6, and F1B hamsters.

Spot no.	Symbol	Protein name	% Cov.	Peptides (95%)	Fold change	
					F1B vs TO-2	F1B vs Bio14.6
725	TF	Serotransferrin	6.4	2	1.26	1.39
1150	OXCT1	Succinyl-CoA:3-ketoacid-coenzyme A transferase 1, mitochondrial	24.2	6	1.25	1.24
1890	ECI	3,2-trans-enoyl-CoA isomerase, mitochondrial	4.5	2	-1.27	-1.20
2066	PRDX2	Peroxiredoxin-2	39.9	4	1.77	1.73
1367	CKB	Creatine kinase B-type	24.7	7	1.23	*1*.*25*
1666	MDH1	Malate dehydrogenase, cytoplasmic	21	5	-1.25	*-1*.*15*
1717	MPST	3-mercaptopyruvate sulfurtransferase	12.1	1	1.24	*1*.*19*
1800	CYC1	Cytochrome c1, heme protein, mitochondrial	16.9	3	1.84	*1*.*33*
1845	TNNI3	Troponin I, cardiac muscle	27.5	6	-1.27	*-1*.*15*
2013	GSTP1	Glutathione S-transferase P	39.1	6	1.34	*1*.*21*
513	VCL	Vinculin	5.3	3	*-1*.*11*	-1.24
1098	EPHX2	Epoxide hydrolase 2	13.4	5	*1*.*05*	1.20
1118	P4HB	Protein disulfide-isomerase	12.6	5	*1*.*08*	1.29
1290	PDHA1	Pyruvate dehydrogenase E1 component subunit alpha, somatic form, mitochondrial	15.1	6	*-1*.*12*	-1.22
1725	LDB3	LIM domain-binding protein 3	62.0	4	*-1*.*19*	-1.25
2104	MYL2	Myosin regulatory light chain 2, ventricular/cardiac muscle isoform	37.4	5	*-1*.*19*	-1.34

% cov.: coverage ratio.

### Expression of identified proteins

**Fig 5:**
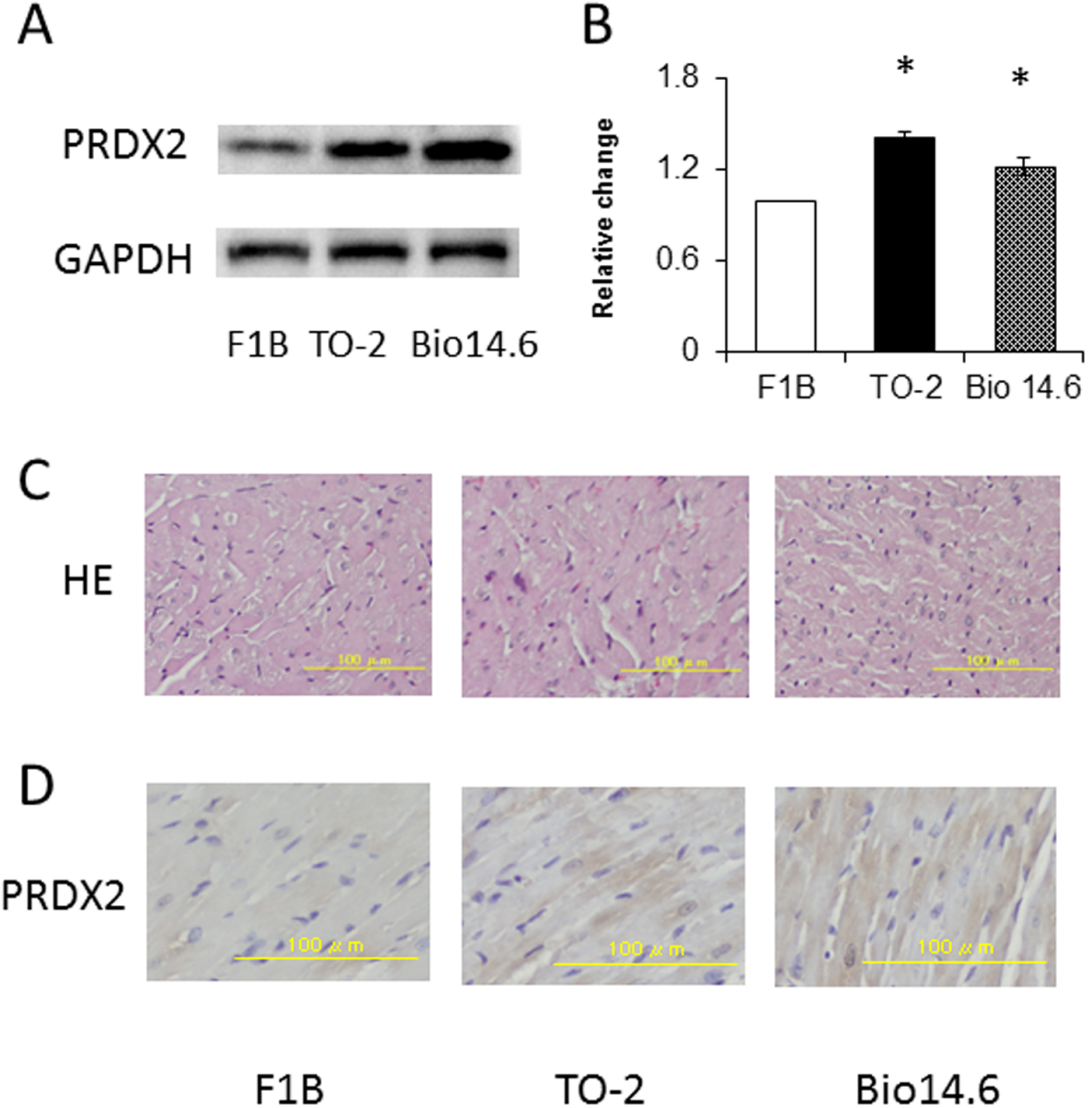
Amounts of PRDX in 6-week-old F1B, TO-2, and Bio 14.6 hamsters. (A) Representative immunoblots of each protein. (B) The amount of each protein quantitated relative to the amount of β-actin and expressed relative to the value of F1B control hamsters. Data are mean±SEM of six animals per group. *P<0.05, versus F1B control hamsters. (C) Light micrographs of myocytes in hematoxylin-eosin stained sections and (D) immunohistochemical staining for PRDX in the left ventricles of representative F1B, TO-2, and Bio 14.6 hamsters. Scale bars, 100 μm.

Because the expression of no. 2066 was significantly higher in both TO-2 and Bio 14.6 hamsters, compared with the F1B control hamsters, western blot analysis was performed to confirm the results of proteomics analysis. The expression of PRDX 2 was significantly up-regulated in TO-2 and Bio 14.6 hamsters, compared with the F1B control hamsters (Fig 5A and 5B). These findings were consistent with those of proteomic analysis. Furthermore, no significant changes in the extent of myocyte hypertrophy in the left ventricle were observed among the TO-2, Bio14.6, and F1B control hamsters (Fig 5C). Examination of immunohistochemically stained LV sections of 6-week-old hamsters showed marked increase in the abundance of PRDX2 in the TO-2 and Bio14.6 hamsters compared with the F1B control hamsters (Fig 5D).

### Functional categories of identified proteins

**Fig 6:**
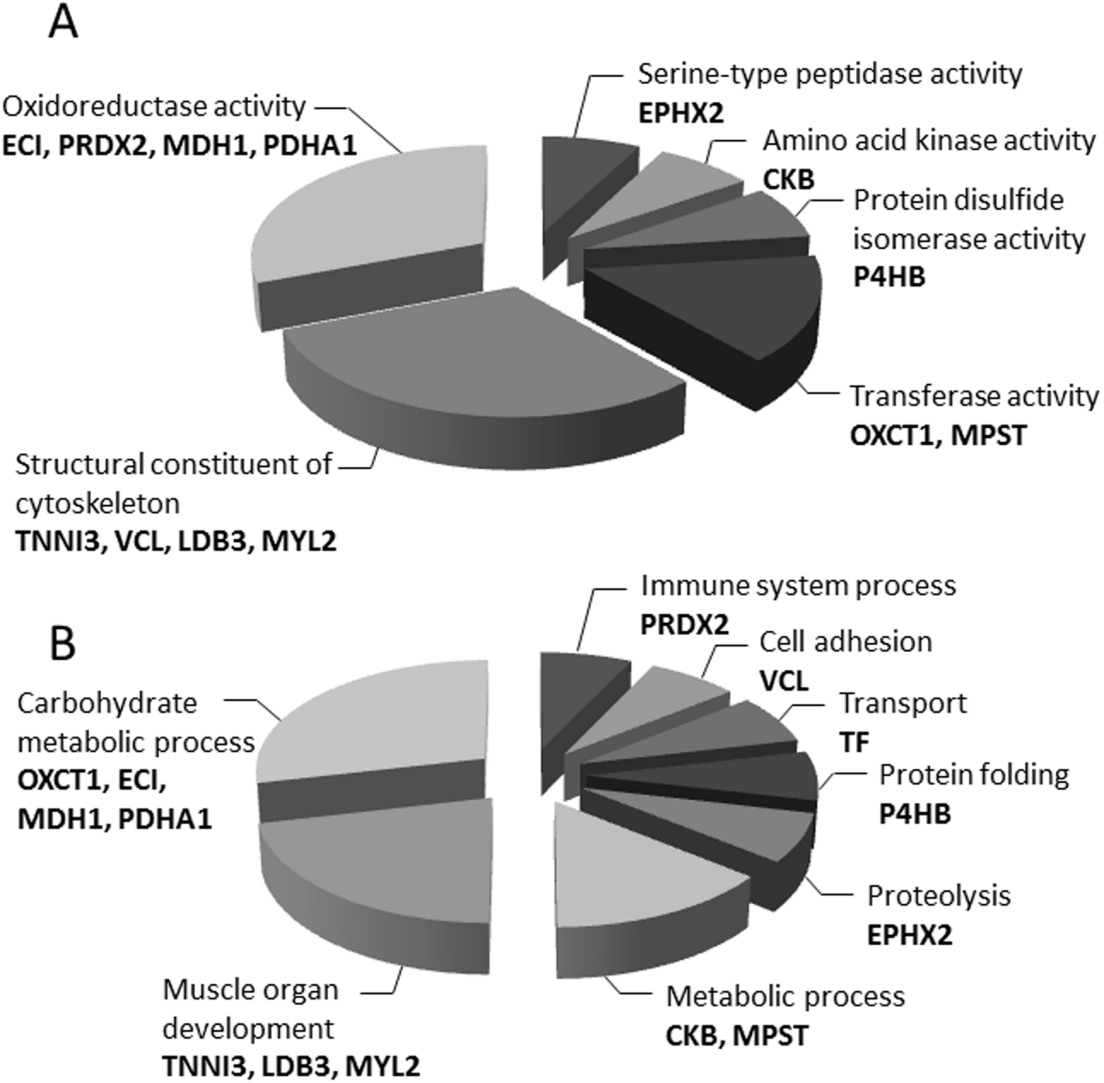
Gene Ontology (GO) annotation of identified proteins. The graphs show the percentages of corresponding GO terms to the total number of annotated proteins. The identified up-regulated and down-regulated proteins were mapped to GO at (A) biological process and (B) molecular function.

To understand their biological roles, the 16 proteins with altered expression in TO-2 and Bio 14.6 hamsters were assigned to protein analysis through an evolutionary relationships (PANTHER) database. Mapping for the molecular function showed that the altered proteins were mainly involved in oxidoreductase activity and structural constituents of the cytoskeleton (Fig 6A). Furthermore, annotation for biological process showed that half of the altered proteins belonged to either the carbohydrate metabolic process or muscle organ development (Fig 6B).

## Discussion

In the present study, PRDX2 was significantly upregulated in 6-week-old TO-2 and Bio 14.6 hamsters. In our previous study on Syrian hamsters (TO-2 strain), we found that diastolic cardiomyopathy manifested severe LV dysfunction after 8 weeks of age, and that plasma troponin T levels were significantly higher at 8 weeks of age than 4, 12, 18, and 32 weeks [14]. Although the values of cardiac functions (LV end-diastolic diameter, LV end-systolic diameter, and LV fractional shortening) and some cardiac phenotype-related gene expression were different at 6 weeks of age, no phenotype manifestation was noted yet. Therefore, our data suggest that PRDX2 is a promising novel marker of early-phase LV impairment in cardiomyopathy.

The PRDX, the antioxidant components of the thioredoxin superfamily [19, 20], have gained recognition as important redox regulating molecules relevant to the mechanisms underlying ischemia-reperfusion injury. There are currently six known prdx enzymes that protect cells and tissues from damage caused by reactive oxygen species in mammals [21, 22]. PRX2 has recently been shown to scavenge H_2_O_2_ generated by platelet-derived growth factor (PDGF) stimulation and negatively regulate the activation of PDGF receptor, a tyrosine kinase receptor [23]. Moreover, it has been shown that PRDX2 is a unique antioxidant in the cardiac system and may represent a potential target for cardiac protection from oxidative stress-induced injury, suggesting the possible effects of PRDX2 in oxidative stress-induced myocyte apoptosis and necrosis [24]. In the present study, the expression of PRDX2 was significantly upregulated in both TO-2 and Bio 14.6 hamsters, compared with F1B control hamsters. Moreover, the present immunohistochemical analysis showed that PRDX2 was expressed in myocytes mainly. PRDX2 has been used to correctly classify mild and severe fibrosis, indicating an anti-oxidative stress reaction during the progress of fibrosis [25]. Given that fibrosis leads to increased myocardial stiffness, thereby promoting cardiac dysfunction, our data suggest that PRDX2 can be potentially used as a new diagnostic and prognostic biomarker of myocardial proteins involved in cardiac impairment. If validated in humans, these biomarkers may facilitate therapeutic interventions that can allow identification of the early phase of LV impairment in cardiomyopathy.

Our study also identified various other biological molecules related to cardiomyopathies. The expression of LDB3 was significantly downregulated in Bio 14.6 hamster only but not in F1B control hamsters. LDB3 is also known as Cypher and Z-band alternatively spliced PDZ-motif (ZASP). The protein encoded by LDB3 interacts with al pha-actinin-2 through its N-terminal PDZ domain and with protein kinase C via its C-terminal LIM domains [26]. LIM-domain-containing cytoskeletal protein knockout mice display disorganized and fragmented Z-discs in both skeletal and cardiac muscles [27]. These mice lead to severe forms of congenital myopathy and cardiomyopathy [28, 29]. Moreover, mutations in its encoding gene have been identified in patients with DCM and HCM [30] by increasing the affinity of the LIM domain for protein kinase C [31]. The results suggest that LDB3 functions as a linker-strut to maintain cytoskeletal structure during contraction. Therefore, downregulation of LDB3 could set marked cardiac hypertrophy reaction.

TNNI3 was significantly under-expressed in TO-2 hamster only but not in F1B control hamsters. This protein is part of the troponin complex that regulates actin-myosin interaction, which plays a key role in the complex contraction-relaxation cycle of striated myofibrils [32]. Advances in molecular genetics of cardiomyopathy have led to the discovery of a large number of mutations in the genes encoding the sarcomeric proteins, such as troponin [33]. Autoantibodies against TNNI3 in a mouse model of DCM, and administration of monoclonal antibodies against TNNI3 induced heart dilation and dysfunction in wild type mice [34]. Other studies showed that altered function of TNNI3 due to either ischemia or depletion of TNNI3 can produce diastolic dysfunction and myocardial hypertrophy [35]. Furthermore, a recent study found that TNNI3 degradation at the C terminus enhanced myofilament Ca ^2+^ sensitivity and slowed force development as well as relaxation [36]. In the present study, the expression of TNNI3 was significantly decreased in TO-2 hamsters only, but not in F1B control hamsters. Cardiac contraction involves sliding and interdigitation of the thick and thin filaments of sarcomeres. Given that contraction is initiated following a rise in cytosolic Ca ^2+^ and its binding to the troponin complex, TNNI3 may contribute to genetic-based diagnosis, risk stratification, and prevention of DCM.

In the present proteomic analysis identified only 4 proteins that showed significant alterations in the left ventricles of both TO-2 and Bio 14.6 hamsters, compared with F1B control hamsters. If the sample number was larger we would detect more number of spots that showed significant difference in both TO-2 and Bio 14.6 hamsters. Previous study demonstrated that the mass and wall thickness of the left ventricles from the Bio 14.6 hamsters were dramatically increased and the LV wall of the TO-2 hamsters was very thin [7]. Our previous study [14] also showed that diastolic cardiomyopathy of the TO-2 hamsters manifested severe LV dysfunction after 8 weeks of age. After the phenotype becomes obvious as cardiac dysfunction or hypertrophy, it is difficult to know whether the identified proteins in left ventricles of cardiomyopathy play a role in the progression of cardiac impairment. Therefore, in the present study we used younger mice (6 weeks of age) whose phenotypes of cardiomyopathy had not been manifested yet. Thus, we believe that the observation of present study suggests that PRDX2 is a promising novel marker of early-phase LV impairment in cardiomyopathy.

## Conclusions

In the present study, we performed proteomic analysis to identify the target molecules associated with heart impairment related to cardiomyopathies in hamsters. Proteomic analysis identified significant alterations in 10 protein spots, with 7 up-regulated and 3 down-regulated proteins in the LV of both TO-2 and Bio 14.6 hamsters, compared with the F1B control hamsters. Our data suggest that PRDX2, a redox regulating molecule, is involved in early-phase left ventricular impairment in hamsters with cardiomyopathy.

## Supporting information

S1 Table List of primers for real-time PCR. (DOCX)
