# Supplementary material for: Proteomics analysis identified peroxiredoxin 2 involved in early-phase left ventricular impairment in hamsters with cardiomyopathy
Source: PLoS One. 2018 Feb 13;13(2):e0192624. doi: 10.1371/journal.pone.0192624 (PMC5810987; doi:10.1371/journal.pone.0192624)
Supplement: S1 Table — (DOCX) [file pone.0192624.s001.docx]

**S1 Table.** List of primers for real-time PCR.

| Gene | Primers | Sequence (5'-3') |
| --- | --- | --- |
| ANP | Forward primer | CAGCAAGCTTCAGATCGTGC |
|  | Reverse primer | GGTTCGCTCCAATATGGCCT |
| BNP | Forward primer | CGGATGGTTCTGTTCCTGCT |
|  | Reverse primer | CGCTCACCTGCATCTTGGAT |
| Collagen I | Forward primer | ATGATGCCAATGTGGTCCGT |
|  | Reverse primer | GGTCAATCCAGTACTCTCCTGAA |
| Collagen III | Forward primer | AGCCACCTTGGTCAGTCCTA |
|  | Reverse primer | AGGACAGATCCGGAGTCACA |
| GAPDH | Reverse primer | TGTTCTAGAGACAGCCGCATC |
|  | Reverse primer | TAACCAGGCGTCCAATACGG |
